# Aztreonam Lysine Increases the Activity of Phages E79 and phiKZ against *Pseudomonas aeruginosa* PA01

**DOI:** 10.3390/microorganisms9010152

**Published:** 2021-01-12

**Authors:** Carly M. Davis, Jaclyn G. McCutcheon, Jonathan J. Dennis

**Affiliations:** Department of Biological Sciences, University of Alberta, Edmonton, AB T6G 2R3, Canada; davis1@ualberta.ca (C.M.D.); jgmccutc@ualberta.ca (J.G.M.)

**Keywords:** bacteriophages, phage therapy, phage–antibiotic synergy, biofilms, *Pseudomonas aeruginosa*, aztreonam

## Abstract

*Pseudomonas aeruginosa* is a pernicious bacterial pathogen that is difficult to treat because of high levels of antibiotic resistance. A promising alternative treatment option for such bacteria is the application of bacteriophages; the correct combination of phages plus antibiotics can produce synergistic inhibitory effects. In this study, we describe morphological changes induced by sub-MIC levels of the antibiotic aztreonam lysine (AzLys) on *P. aeruginosa* PA01, which may in part explain the observed phage–antibiotic synergy (PAS). One-step growth curves for phage E79 showed increased adsorption rates, decreased infection latency, accelerated time to lysis and a minor reduction in burst size. Phage E79 plus AzLys PAS was also able to significantly reduce *P. aeruginosa* biofilm growth over 3-fold as compared to phage treatment alone. Sub-inhibitory AzLys-induced filamentation of *P. aeruginosa* cells resulted in loss of twitching motility and a reduction in swimming motility, likely due to a reduction in the number of polar Type IV pili and flagella, respectively, on the filamented cell surfaces. Phage phiKZ, which uses Type IV pili as a receptor, did not exhibit increased activity with AzLys at lower sub-inhibitory levels, but still produced phage–antibiotic synergistic killing with sub-inhibitory AzLys. A one-step growth curve indicates that phiKZ in the presence of AzLys also exhibits a decreased infection latency and moderately undergoes accelerated time to lysis. In contrast to prior PAS studies demonstrating that phages undergo delayed time to lysis with cell filamentation, these PAS results show that phages undergo accelerated time to lysis, which therefore suggests that PAS is dependent upon multiple factors, including the type of phages and antibiotics used, and the bacterial host being tested.

## 1. Introduction

*Pseudomonas aeruginosa* is a Gram-negative opportunistic pathogen that possesses extreme multi-drug resistance (MDR) and is commonly acquired in hospital settings [1]. Cystic fibrosis (CF) patients are highly susceptible to lung infections caused by a variety of bacteria, but *P. aeruginosa* is the most prevalent pathogen causing infection in adult CF patients, and is one of the leading causes of morbidity and mortality in the CF community [2,3]. This is due to intrinsic MDR and adaptability of pathogenic *P. aeruginosa*, including the ability to establish biofilms that lead to chronic infection [4,5,6]. Of CF individuals infected with *P. aeruginosa,* 16.9% of them have MDR infections (3). Individuals with CF are especially susceptible to lung infections due to mutations in the cystic fibrosis transmembrane conductance regulator (CFTR) gene encoding a chloride anion channel; impairment of its function disrupts salt and water transport in the lungs and leads to the build-up of thick, sticky mucus. The inability of CF patients to clear this sticky mucus from their lungs compromises one of their primary innate defense mechanisms against bacterial infection in human airways [7]. While the expected lifespan of individuals with CF has risen over the past few decades due to improved therapeutic regimens, irreversible lung damage due to bacterial colonization continues to lead to decreased pulmonary function and premature death [8]. The development of therapeutic alternatives to eliminate *P. aeruginosa* and other MDR bacteria from the lungs of individuals with CF is imperative.

One alternative treatment option for MDR infections is “phage therapy”, the clinical use of bacteriophages, also known as phages. Phages are bacterial viruses that exclusively attack and lyse specific host bacteria, leaving beneficial bacterial flora unharmed [9]. Phage therapy has been used in some Eastern European countries for over a century but was largely abandoned in the West in the 1940s with the advent of antibiotics [9]. The misuse of antibiotics, however, has led to an increase in antibiotic resistance, with the prediction that MDR bacterial infections will cause over 10 million deaths by 2050 worldwide if left unchecked [10]. Because of this threat, acceptance of phage therapy as a potential treatment option is growing, and the first North American phage therapy center, the Center for Innovative Phage Applications and Therapeutics (iPATH), opened at the University of California San Diego in June 2018. In addition to high host specificity, phages are advantageous for therapy because they use different mechanisms to kill bacteria than antibiotics, meaning they are effective at clearing MDR bacterial infections [11]. As well, some phages possess the ability to penetrate and/or break down bacterial biofilms that antibiotics often show decreased effectiveness against due to the increased antibiotic resistance exhibited by cells within these communities [11]. While phages hold many benefits, selection of bacteriophages for therapy must be made carefully, as some phages can also increase bacterial virulence through lysogenic conversion and be maintained through cell division as a prophage [12]. Additionally, bacteria can develop resistance to phage infection by several mechanisms including the mutation of receptors on their cell surface, leaving phages unable to adsorb to and kill the bacterial cell [9]. The use of phage cocktails can overcome this problem by combining phages that target different cell surface receptors [9].

It has been shown by a variety of studies that some antibiotics can induce increased phage activity at sub-inhibitory concentrations, a phenomenon termed “phage–antibiotic synergy” (PAS) [13,14,15,16,17]. This natural phenomenon was described following the discovery that some phage titers and plaque sizes increase in the presence of sub-inhibitory levels of antibiotics [13]. Lytic phage activity was shown to increase in the presence of four different classes of antibiotics against *Burkholderia cenocepacia,* even with cells possessing elevated antibiotic resistance, suggesting that PAS could still be used as an alternative treatment option for MDR bacterial infections [14]. The well-studied bacteriophage T4 has been shown to have both increased activity in the presence of cefotaxime, as well as cause increased susceptibility to the antibiotic by the host *Escherichia coli* [15]. Additionally, it has been shown that the combined application of phage and antibiotic treatments is significantly more effective at eradicating *P. aeruginosa* biofilms than either treatment alone [16]. It has been speculated that phages and antibiotics likely evolved synergistic interactions because together they reduce the chances that bacterial populations will develop resistance to either killing agent [18].

With many pharmaceutical companies currently reducing the research and development of new antibiotics because of a lack of return on investment [19], combined antibiotic and phage treatment may be one way of increasing the efficacy of antibiotics that are ineffective on their own. Therefore, the objective of this study was to determine whether a synergistic effect exists between the virulent phage E79 and the antibiotic aztreonam lysine (AzLys) [20] on *P. aeruginosa* strain PA01 in vitro. Trend et al. [21] showed that phage E79 does not induce apoptosis or the production of inflammatory cytokines on airway epithelial cells isolated from children with or without CF. They also consider the potential of E79 as a phage therapy agent due to its wide host range against *P. aeruginosa* clinical isolates, stable high-titer stocks, and no known presence in bacterial pathogenicity islands [21]. Our results extend these previous findings and identify a PAS effect of increased time to lysis, increased infection efficiency and plaque sizes by E79 in the presence of AzLys, as well as the increased destruction of biofilms. Concurrently, AzLys also negatively affects the function of virulence factors Type IV pili (T4P) and flagella of *P. aeruginosa* but is able to increase the killing activity of phage phiKZ, which uses the T4P as its cellular receptor. Our results show that the combined use of phages E79 or phiKZ with AzLys has increased efficacy in vitro against *P. aeruginosa* PA01 and presents a promising alternative treatment option for chronic *P. aeruginosa* infections.

## 2. Materials and Methods

### 2.1. Bacteria, Phage, and Growth Conditions

*P. aeruginosa* strain PA01 [22] was used in all experimental analyses. Bacteriophage E79 is a lytic phage that belongs to the *Myoviridae* family and infects many *P. aeruginosa* strains [23,24]. E79 was obtained from the Félix d’Herelle Reference Center for Bacterial Viruses (Laval University, Quebec, Canada). PhiKZ is a jumbo lytic phage that belongs to the *Myoviridae* family and infects many *P. aeruginosa* strains [25] and was gifted to us by Joe Bondy-Denomy (University of California, San Francisco, USA). Propagation of E79 and phiKZ phage stocks and determination of titer were performed on PA01 using the double agar overlay method as previously described [26,27]. Phage titers used in each experiment are described in the relevant sections. Bacteria were grown aerobically overnight at 37 °C on half-strength Lennox (½ LB) solid medium or in ½ LB broth with shaking at 225 rpm. PA01 overnights were subcultured 1:100 and grown to an optical density at 600 nm (OD_600_) of 0.1 to obtain exponential-phase growth cultures. In all experiments, AzLys was added to media in the commercially available soluble form of Cayston (Gilead Inc., Foster City, CA, USA), which is an aztreonam derivative that improves respiratory symptoms of CF patients caused by chronic *P. aeruginosa* infections [20]. Statistical analysis for all experiments was conducted using GraphPad Prism 8 (Graph-Pad Software Inc., San Diego, CA, USA).

### 2.2. Minimum Inhibitory Concentration (MIC)

MIC protocol was followed according to Wiegand et al. [28] using ½ LB broth and the antibiotics were prepared either in 0.17% saline (AzLys) or in 100% ethanol (aztreonam) and diluted to the desired concentrations in ½ LB broth. The OD_600_ was measured after 24 h using a Victor X3 spectrophotometric plate reader (PerkinElmer, Woodbridge, ON, Canada). The experiment was repeated in biological triplicate, with three replicates each.

### 2.3. Transmission Electron Microscopy (TEM)

*P. aeruginosa* PA01 cells were prepared for electron microscopy as follows. A volume of 100 μL of a 0.2475 mg/mL AzLys stock, or 0.17% NaCl for the control, was mixed with 3 mL of 0.7% ½ LB top agarose and poured onto a ½ LB agar plate to obtain a final concentration of 1.06 μg/mL, assuming AzLys fully diffuses. Once solidified, 100 μL of exponential-phase PA01 culture was spread gently across the top agarose and incubated at 37 °C overnight. Cells were collected from control and AzLys plates, standardized by weight, suspended in 1 mL 1 × phosphate-buffered saline (PBS), pH 7.2, washed twice with 1 mL of 1 × PBS, resuspended in 200 μL of EM fixative (2.5% glutaraldehyde, 2% paraformaldehyde, 0.1 M phosphate buffer, pH 7.2) and incubated at room temperature for 30 min. The fixed cells were pelleted at 8000× *g* for 1 min and resuspended in 1 mL of 1 × PBS. For visualization of bacterial samples, 10 μL of sample was incubated on a carbon-coated copper grid for 2 min and stained with 2% phosphotungstic acid (PTA) for 10 s. To visualize phage interaction with PA01, E79 was propagated as described above, substituting ½ LB agarose for overlays. Bacterial samples were mixed in a 1:2 ratio with high-titer 10^9^ PFU/mL E79 stock for 2 min and 10 μL of this mixture was loaded on a carbon-coated copper grid as described above. Transmission electron micrographs were captured using a Philips/FEI (Morgagni) transmission electron microscope with charge-coupled device camera at 80 kV (University of Alberta Department of Biological Sciences Advanced Microscopy Facility, Edmonton, AB, Canada). The average length and width measurements ± standard deviation for PA01 were calculated using Microsoft Excel from 10 individual bacteria measured using ImageJ software [29] (NIH, Bethesda, MD, USA).

### 2.4. Phage–Antibiotic Synergy Modified Double Agar Overlay

Double agar overlay plaque assays were used to determine the effect of AzLys on plaque size [26] with modifications [14]. Briefly, 100 μL of exponential-phase PA01 culture was incubated with 100 μL of phage stock at 10^3^ PFU/mL for 7 min, mixed with 100 μL of AzLys and 3 mL of 0.4% ½ LB top agar, and overlaid onto 1.5% agar ½ LB solid media. Plates were incubated at 37 °C overnight until plaques formed. For controls, 100 μL of modified suspension medium (SM) (50 mM Tris-HCl pH 7.5, 100 mM NaCl, 10 mM MgSO_4_) was added in place of each AzLys concentration or phage suspension to account for agar dilution effects. AzLys concentrations used were calculated as 1.875, 3.75, 7.5 and 10 μg/mL within the 3 ml layer of top agar. Expecting the AzLys to diffuse evenly throughout the 20 mL agar plate, the final experimental concentrations are 0.27, 0.53, 1.06 and 1.41 μg/mL, respectively. Phage plaques were backlit and viewed under the magnifying glass of a New Brunswick Scientific colony counter (model C110), and plaque diameter was measured using digital calipers manufactured by Tresna (Guilin, China). Ten plaques were measured at random per treatment group. Experiments were repeated in biological and technical triplicate and average plaque diameter was calculated from the 90 plaque measurements. Data were compared using an unpaired one-way ANOVA with Dunnett’s post-test.

### 2.5. Phage Plaquing Assays

Phage plaquing ability was determined as described previously [27], with slight modifications. Briefly, 100 µL of overnight culture was mixed with either 200 µL ½ LB or 100 µL AzLys and 100 µL ½ LB for a desired concentration of 1.06 μg/mL AzLys. This concentration was used because it is in the middle of the three concentrations that showed PAS activity for E79. A volume of 3 mL of 0.7% ½ LB top agar was added to this and overlaid onto ½ LB plates and allowed to dry at room temperature for 1 h. Phage stocks were 10-fold serially diluted in SM. A volume of 5 µL of each dilution was spotted onto the plates and incubated overnight at 37 °C.

### 2.6. One-Step Phage Growth Curve

The bacteriophage growth curves were performed as previously described [30] with some modifications. Briefly, two 1:100 subcultures were grown in the absence or presence of 1.06 μg/mL AzLys to an OD_600_ of 0.1, equal to approximately 2.0 × 10^8^ CFU/mL. This concentration of AzLys was used because it is in the middle of the three concentrations that showed PAS activity for E79. Phages were added to 10 mL of each exponential-phase culture to obtain a multiplicity of infection (MOI) of three and incubated at 37 °C with aeration at 225 rpm. A volume of 10 μL was removed in triplicate from each culture at desired time points, and serially diluted in 90 μL of chilled 1 × PBS. A volume of 5 μL of each dilution was spotted on ½ LB double agar overlays containing PA01 culture to assess phage titer. This process was repeated in triplicate, and the rate of phage adsorption was calculated as the change in PFU/mL over time during the latent period. Burst size was calculated using the formula “burst size = P − x/I − x” where P is the maximum number of phages after lysis, I is the number of phages initially added to the culture, and x is unadsorbed phage. Statistical analysis was performed using a paired *t*-test.

### 2.7. Twitching Motility Assay

*P. aeruginosa* PA01 twitching motility in the presence of AzLys was assessed as previously described [31], with some modification. A single colony was suspended in 100 μL of ½ LB broth and stab inoculated with a sterile toothpick through a 3 mm thick ½ LB 1% agar layer containing 0, 0.27, 0.53, 1.06, or 1.41 μg/mL of AzLys. Plates were incubated with humidity at 37 °C for 24 h. Twitching zones beneath the agar were stained using 3 mL of TM developer solution (40% water, 10% glacial acetic acid, 50% methanol) for 30 min and measured using ImageJ software [29] (NIH, Bethesda, MD, USA). Experiments were repeated in biological and technical triplicate and average twitching diameter was calculated from the nine twitching zones. Statistical analyses were performed using an unpaired one-way ANOVA and Dunnett’s post-test.

### 2.8. Swimming Motility Assay

Swimming motility of PA01 in the presence of AzLys was assessed as previously described [32] with some modifications. Briefly, 100 mL/plate ½ LB 0.3% agar containing 0, 0.27, 0.53, 1.06, or 1.41 μg/mL of AzLys was poured 7 mm thick in 15 mm petri dishes and allowed to solidify for 2 h at room temperature. A volume of 2 μL of overnight PA01 liquid culture standardized to an OD_600_ of 1.0 was injected halfway through the agar. The plates were incubated for 24 h at 37 °C and swimming zones were measured using ImageJ software [29] (NIH, Bethesda, MD, USA). Experiments were repeated in biological and technical triplicate and average swimming diameter was calculated from the nine swimming zones. Statistical analyses were performed using an unpaired one-way ANOVA and Dunnett’s post-test.

### 2.9. PAS Killing Assay

In a 96-well microtiter plate, 100 μL of exponential-phase PA01 culture at 2 × 10^8^ CFU/mL was added to 50 μL of phiKZ phage stock for the desired MOI and 50 μL of AzLys or ½ LB to test PAS effects. For controls, 100 μL of SM was added in place of phage suspension, and ½ LB was added in place of AzLys. The plates were incubated for 24 h at 37 °C and 225 rpm. The OD_600_ was measured using a Victor X3 spectrophotometric plate reader (PerkinElmer, Woodbridge, ON, Canada). Each treatment was repeated in biological triplicate, with three replicates each. Statistical analyses were performed using unpaired *t*-tests.

### 2.10. PA01 Biofilm Formation and PAS Treatment

Static biofilms were formed on polystyrene pegs of the MBEC™ biofilm inoculator with a 96-well base (Innovotech Inc., Edmonton, AB, Canada) following established protocols (MBEC™ Assay Procedural Manual, Version 1.1., 2015), with some modifications. *P. aeruginosa* PA01 overnight cultures were grown at 37 °C in ½ LB broth for 18 h, standardized, then diluted 1:100 in tryptic soy broth (TSB) to obtain a starting inoculum of approximately 10^7^ CFU/mL. The 60 central wells of the microtiter plate were filled with 200 μL of starting inoculum and the outside wells were filled with 200 μL of TSB broth as aseptic controls and to prevent edge effects due to evaporation. The plates were covered with 96-peg polystyrene lids and incubated for 24 h at 37 °C and 120 rpm with humidity.

The developed PA01 biofilms were washed in 200 μL of 0.9% saline for 5 min to remove non-adherent cells and the lid was transferred to a treatment plate containing 0, 1.41, 1.6, 1.8, or 2.0 μg/mL of AzLys with and without 10^4^ PFU E79 in 200 μL volume total. The biofilms were incubated for 4 h at 37 °C and 120 rpm with humidity followed by washing in 200 μL of 0.9% saline for 5 min. To quantify the biofilm biomass, the lid was transferred to a 96-well plate containing 200 μL of 0.9% saline and biofilms were dislodged from the pegs using a water bath sonicator (Branson Ultrasonic Cleaner Model B-32) for 30 min. The OD_600_ was measured using a Victor X3 spectrophotometric plate reader (PerkinElmer, Woodbridge, ON, Canada). Each treatment was repeated in biological triplicate, with six replicates each.

### 2.11. Scanning Electron Microscopy (SEM) of Biofilms

To visualize the morphology of *P. aeruginosa* PA01 biofilms, MBEC™ pegs carrying biofilms grown for 24 h and treated with 0 or 1.6 μg/mL of AzLys for 4 h were broken off the lid using sterile flat nose pliers and prepared as follows. Biofilm pegs were suspended in EM fixative overnight at room temperature, then washed three times in 1 × PBS for 10 min each. The fixed samples were dehydrated using a series of ethanol washes for 10 min each (50, 70, 90, 100, 100%) followed by ethanol:hexamethyldisilazane (HMDS) washes (75:25, 50:50, 25:75) for 10 min each. Finally, the biofilms were washed with 100% HMDS for 20 min, and then left in HMDS overnight with the lid slightly ajar to air dry. HMDS was used in place of critical point drying [33]. Once dried, the samples were mounted onto SEM stubs, sputter coated with gold and viewed in a Zeiss Sigma Field Emission SEM (University of Alberta Department of Earth and Atmospheric Sciences Scanning Electron Microscope Laboratory, Edmonton, AB, Canada).

## 3. Results and Discussion

### 3.1. Effects of Sub-Inhibitory AzLys on PA01 Morphology

**Figure 1:**
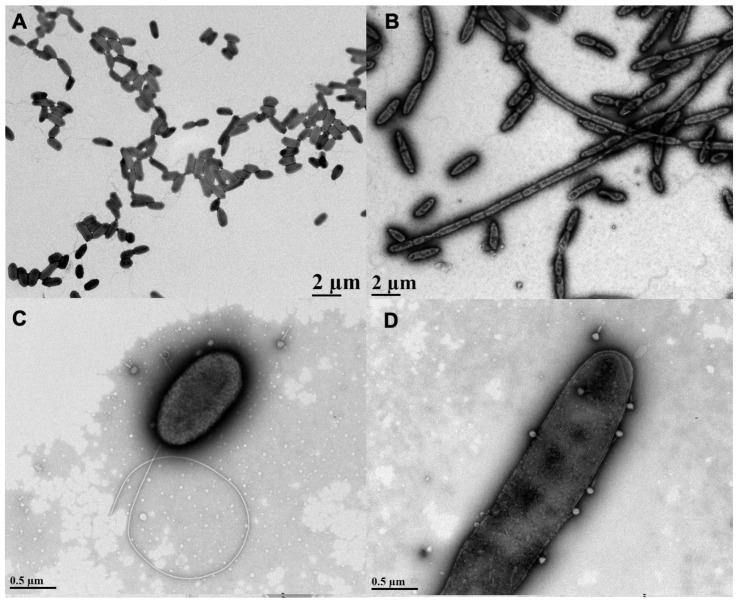
Effects of sub-inhibitory aztreonam lysine (AzLys) on *Pseudomonas aeruginosa* PA01 morphology and phage E79 recognition. Electron micrographs showing PA01 grown under normal conditions (**A**) without or (**C**) with E79, and (**B**) PA01 grown in the presence of 1.06 μg/mL AzLys (**B**) without or (**D**) with E79. Bacteria and phages were stained with 2% PTA and visualized by transmission electron microscopy at 4,400-fold (**A**,**B**) and 28,000-fold (**C**,**D**) magnification. Images are representative of at least 10 different bacterial cells per sample.

AzLys belongs to the monobactam class of antibiotics that inhibit cell wall biosynthesis, and many antibiotics that disrupt cell division have been observed to cause morphological changes at sub-inhibitory concentrations [13,14,34,35,36]. To investigate whether AzLys changes the morphology of *P. aeruginosa* PA01, we observed PA01 grown in the presence or absence of a sub-inhibitory concentration of AzLys using transmission electron microscopy (TEM). The MIC of AzLys for the PA01 strain tested is 8 μg/mL and the sub-inhibitory concentration is <2 μg/mL (Supplementary Materials Figure S1). To show that *P. aeruginosa* strain PA01 is not unusual in its resistance profile to AzLys, its MIC was compared to other *P. aeruginosa* strains (Supplementary Materials Figure S2; Supplementary Materials Table S1). The sub-inhibitory concentration is defined as the point where no bacteria are killed by the antibiotic concentration present. In the presence of AzLys, the morphology of PA01 is filamentous, a dramatic change from its wild-type single-cell rod structure (Figure 1A,B). These bacteria appear unable to form a septum between cells to complete cell division, forming significantly larger cells in both diameter and length under the stress of AzLys. Under normal growth conditions, single PA01 cells are on average 1.15 ± 0.18 μm in length and 0.51 ± 0.05 μm in width. These dimensions increase significantly in the presence of AzLys, with single PA01 cells (i.e., cells that have not undergone filamentation) measuring 1.8 ± 0.18 μm in length (*p* < 0.0001) by 0.55 ± 0.03 μm in width (*p* < 0.05). This morphological change could aid phage activity by a number of potential mechanisms. It has been suggested that an aberrant filamentous cell structure may cause increased bacterial membrane sensitivity to phage lysis proteins, such as lysozymes and holins, as well as allow for an increased rate of phage production [13]. This increased rate of phage production is correlated with cell size and is proportional to the amount of protein-synthesizing machinery within a cell at the time of infection, leading to increased phage burst sizes [37]. An increased burst size would allow low numbers of initial phages to self-propagate faster than normal under the same conditions. Additionally, it was observed that phage adsorption is directly proportional to cellular surface area [37]; therefore, increased cell surface area upon AzLys-induced filamentation may result in an increased rate of phage adsorption and allow phages to infect and spread through a bacterial population more quickly.

The lytic phage E79 uses LPS as a receptor to bind to its bacterial host for infection [38] and any increase in cell membrane will likely result in an increase in LPS, as it is a major component of the outer membrane of Gram-negative bacteria. To observe whether there may be an increased association of E79 with filamentous cell surfaces, a high-titer E79 stock was incubated with PA01 bacterial samples grown in the presence or absence of sub-inhibitory concentrations of AzLys and viewed by TEM. An increase in the number of phages congregated on the surface of filamentous PA01 bacterial cells grown in the presence of AzLys was observed compared to the number of E79 adhered to normal PA01 bacterial cells (Figure 1C,D). Based on 10 different TEM pictures, there was an average of 2 ± 1 phage per cell under normal growth conditions, and 5 ± 2 phage per cell in the presence of AzLys (data not shown). These results show that filamentous cells are larger and can attract more phage per cell than normal growing PA01 cells. These observations are consistent with reported findings that elongated or filamentous cells may provide phages with increased access to their receptors on the cell surface, therefore promoting increased phage production and/or accelerated time to lysis [14,37].

### 3.2. Effects of Different Sub-Inhibitory AzLys Concentrations on E79 Activity

**Figure 2:**
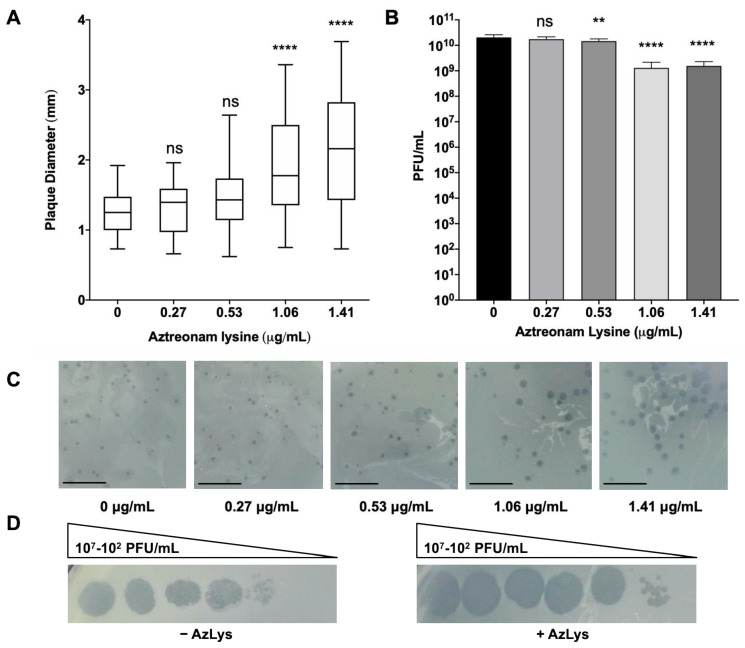
E79 and aztreonam lysine (AzLys) exhibit phage–antibiotic synergy (PAS) on *P. aeruginosa* PA01. (**A**) Box plot of plaque diameter, and (**B**), bar chart of average phage titer, of phage E79 in the presence of different concentrations of sub-inhibitory AzLys determined by a modified double agar overlay assay. Results are from three separate trials, with error bars representing the (**A**) largest and smallest plaque diameter and (**B**) standard deviation. Statistical analysis was performed using a one-way ANOVA with Dunnett’s post-test compared to the control (**, *p* < 0.01; ****, *p* < 0.0001; ns, not significant). (**C**) Visual representation of E79 plaque morphology at increasing AzLys concentrations. All images were photographed at identical magnifications. Scale bars equal 20 mm. (**D**) Infection efficiency of E79 in the absence (-) or (+) presence of 1.06 μg/mL AzLys. Images are representative of three biological replicates, each with three technical replicates.

To determine whether the presence of sub-inhibitory concentrations of AzLys could increase the activity of E79 on *P. aeruginosa* PA01, plaque sizes were compared in a double agar overlay assay in the presence or absence of sub-inhibitory AzLys concentrations (Figure 2A,C). Because plaque size is largely determined by burst size and time to cell lysis [13], increased plaque size is representative of increased phage lytic activity. Our results show a significant increase in the average plaque diameter of E79, producing plaques 1.7-fold larger in the presence of 1.41 μg/mL and 1.6-fold larger for 1.06 μg/mL AzLys as compared to the control (*p* < 0.0001). No significant increase in plaque size was observed for the two lower AzLys concentration of 0.27 and 0.53 μg/mL. Previous studies show that antibiotics affect bacterial morphology and/or growth rate, allowing the lytic activity of phages to increase [13,14,34,35,36,37]. Our results therefore suggest that there is a minimum concentration of antibiotic necessary to cause a physiological change in the bacterium and induce increased phage activity. Phage production was affected minimally, with a small decrease in titer observed at 0.53 (*p* < 0.01), 1.06 and1.41 μg/mL (*p* < 0.001) of AzLys (Figure 2B). 

A plausible explanation for the small reduction in titer is discussed in Section 3.3. These results show that AzLys has a concentration-dependent effect on E79 activity; plaque sizes increase in diameter as sub-inhibitory AzLys concentrations increase (Figure 2A,C). Similar results have been observed in *B. cenocepacia, Staphylococcus aureus*, and *E. coli*, which show an increase in phage plaque sizes as the sub-inhibitory concentration of various antibiotics is increased [14,15,39]. However, it has been demonstrated that cell size was not the only determinant of plaque size when comparing the effects of different antibiotics at sub-inhibitory concentrations [35]. The observed effects of AzLys may be due to more than one mechanism indirectly linked to increased cell size as described above.

In addition to plaque size and phage titer, infection efficiency of E79 on PA01 changes in the presence of AzLys (Figure 2D). Efficiency of infection is increased by around 10-fold in the presence of 1.06 μg/mL of AzLys, the zone of lysis appears to be larger at all dilutions compared to the control, and plaque size is increased at the lowest dilution. The results suggest that E79 is able to infect PA01 cells better in the presence of AzLys. These results support our hypothesis that a one-way synergistic relationship exists between E79 and AzLys; the presence of sub-inhibitory concentrations of AzLys induces an increase in E79 activity as observed by the increase in E79 plaque size and infection efficiency on PA01.

### 3.3. Effects of AzLys on E79 Phage Growth Curve

**Figure 3:**
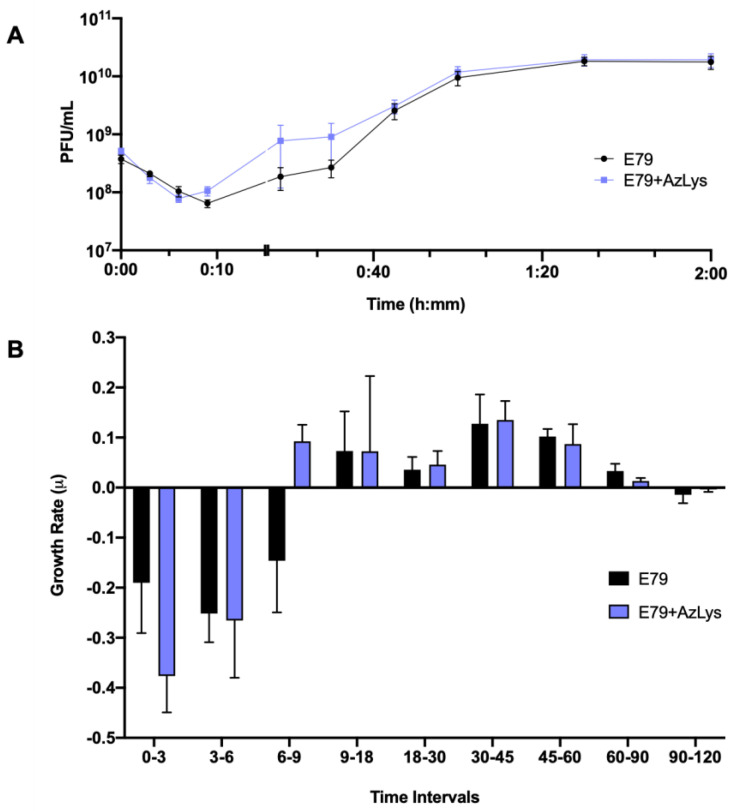
Aztreonam lysine (AzLys) shortens the latent period of the E79 one-step growth curve. Phage E79 was mixed with *P. aeruginosa* PA01 grown with or without 1.06 μg/mL AzLys at a MOI of approximately three. Phage titer was determined periodically over a 2 h incubation. (**A**) The one-step growth curve of E79, with each point representing the average of nine samples from three separate trials. Statistical analysis was performed using a paired *t*-test. (**B**) Growth rate of graph in **(A)** determined using the averages from each trial and calculated using growth rate equation log10N-log10N0 = (μ/2.303) (t-t0). All error bars represent the standard error of the mean (SEM).

To determine what aspects of E79 lifecycle are being affected, we performed a one-step phage growth curve for E79 on PA01 in the presence or absence of 1.06 μg/mL AzLys. The phage growth curves are similar at certain time points of the infection cycle but differ in a number of ways when its host is grown in the presence of sub-inhibitory AzLys (Figure 3A,B). Firstly, there is a reduction in the latency period from approximately nine to six min (Figure 3A). This is shown again in Figure 3B, where phage growth rate has been calculated for each time range and is increasing at time interval 6–9 in the presence of AzLys, while still decreasing under normal conditions. Though this is a short time frame, it should be noted that a consistent decline in phage titer is observed every 3 min prior to these measurements (intervals 0–3 and 3–6), and is still declining in the absence of AzLys (interval 6–9) (Figure 3A,B). Secondly, the decreased latent period is followed by moderate accelerated time to lysis between 9 and 45 min, though the rates of growth are not substantially different (Figure 3B). Despite the initial acceleration, lysis begins to slow, and the remainder of the time points do not differ between the two curves. The E79 + AzLys cycle is not completed earlier, and the total phage production following a single cycle of phage growth is not different between the two treatments. Given how similar many of the time points are between the two curves, the differences between the two curves are noteworthy. Using a paired *t*-test, the two curves compared to each other are statistically different from one another (*p* < 0.05), but there is no statistically significant difference in titer seen at individual time points. Lastly, the average rate of phage adsorption increases from 5.8 × 10^5^ to 1.2 × 10^6^ PFU mL^−1^ sec^−1^ in the presence of AzLys (*p* = 0.0035), supporting our hypothesis that E79 has increased association with the cellular surface in the presence of AzLys.

In addition to providing information about phage adsorption and latency period, burst size can also be calculated from phage growth curves [39]. E79 burst size decreases from 58 to 44 phage per cell in the presence of AzLys, possibly explaining the slight reduction seen in phage titer after overnight infection in Figure 2B. A decrease in latency period shortens the amount of time for phage maturation, which can result in reduced burst size, but can also increase the phages’ rate of exponential growth [40]. Though most PAS studies that examine phage production typically see an increase in burst size or final phage titer [13,14,15,34,36,41,42,43,44], it is important to note that PAS has still been shown to occur in the absence of increased phage production [14,35]. This discrepancy in phage production highlights that PAS may be generated by many different aspects of the phage infection cycle, and as proposed previously [14], it is possible that many different molecular mechanisms are responsible for the total PAS effects observed for every different phage and antibiotic combination. A study by Kim et al. (2018) reported that increased T4 phage production in *E. coli* with the addition of ciprofloxacin is a result of delayed time to lysis caused by an insufficiency of holins in the larger filamented bacteria [17]. In the present study, the observed PAS in the presence of AzLys, with unchanging final phage production after one phage infection cycle, may be explained by the observed accelerated time to lysis rather than a delayed time to lysis. Our growth curve results support the conclusion that the increased PAS lytic activity of E79 in the presence of AzLys may be caused by a combination of increased phage adsorption, potentially due to cell filamentation, and accelerated time to lysis.

### 3.4. PAS Treatment of PA01 Biofilms In Vitro

**Figure 4:**
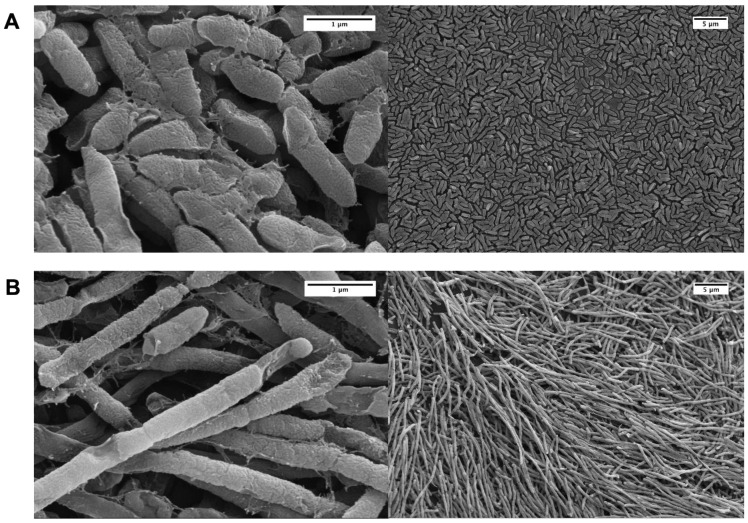
Effect of sub-inhibitory aztreonam lysine (AzLys) on *P. aeruginosa* PA01 biofilm morphology. Electron micrographs show PA01 biofilm under (**A**) normal conditions or (**B**) in the presence of 1.6 μg/mL AzLys. Biofilm pegs were mounted onto stubs, sputter coated with gold, and visualized using a Zeiss Sigma Field Emission SEM at 20,000-fold (left panels) and 2000-fold (right panels) magnification.

Recently, a number of papers have been published that examine different aspects of PAS on *P. aeruginosa* biofilms [16,45,46,47,48,49,50,51,52,53]. We were particularly interested to discover whether pragmatic PAS with a lytic phage and AzLys was more effective at eradicating PA01 biofilms than phage treatment alone. To test this hypothesis, we used the MBEC™ biofilm inoculator with 96-well base plates (Innovotech Inc., Edmonton, AB, Canada) to create identical biofilms growing on plastic pegs. Because biofilms are different from planktonic cells on transcriptomic, proteomic and physiological levels [54] we first sought to determine whether AzLys affects PA01 biofilms similarly to the effects observed on planktonic PA01 cells. Consistent with observations that biofilms exhibit increased resistance to antibiotics, we were able to increase the maximum sub-inhibitory concentration from 1.41 μg/mL to 2 μg/mL AzLys without inhibiting cell growth (data not shown). PA01 biofilms grown for 24 h and treated for 4 h in the presence or absence of 1.6 μg/mL AzLys were imaged using scanning electron microscopy (SEM) (Figure 4). Similar to planktonic PA01, AzLys causes aberrant division of biofilm cells and results in long filamentous structures, suggesting that PAS effects could also be observed with biofilm-grown cells.

**Figure 5:**
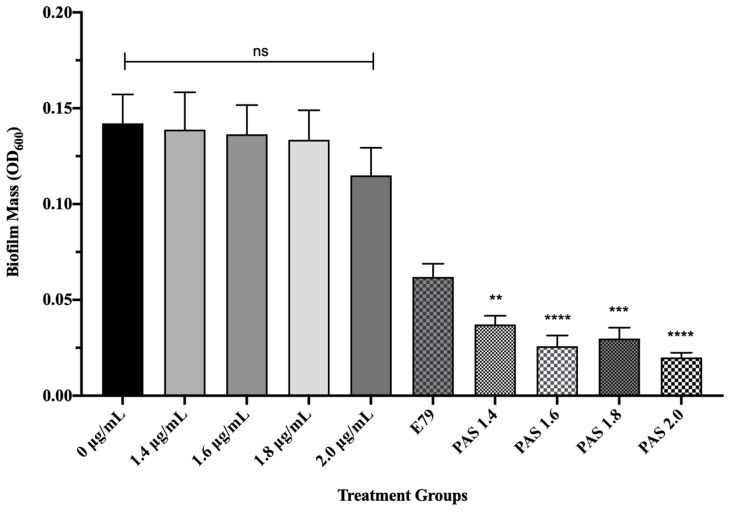
E79 and aztreonam lysine (AzLys) exhibit phage–antibiotic synergy (PAS) on *P. aeruginosa* PA01 biofilms. Biofilms were grown for 24 h followed by treatment for four hours in TSB media at 37 °C with shaking at 120 rpm with added sub-inhibitory AzLys, E79 phage (10^4^ PFU/well), or a combination of both. Biofilms were disrupted back into a planktonic culture using a water bath sonicator, and optical density at 600 nm (OD_600_) was measured to obtain biofilm mass. Statistical analysis was performed using ANOVA and Dunnett’s post-test compared to the control (**, *p* < 0.01; ***, *p* < 0.001; ****, *p* < 0.0001). Error bars represent the standard error of the mean (SEM).

To test whether AzLys causes E79 to be more effective at eradicating PA01 biofilms than treatment with E79 alone, 60 identical PA01 biofilms were grown for 24 h and treated for 4 h with or without varying concentrations of AzLys (1.4; 1.6; 1.8; 2 μg/mL) and E79 (10^4^ PFU/well). The average PA01 biofilm mass (OD_600_) plus or minus the standard deviation for the 10 different treatment groups is shown in Figure 5. No statistically significant difference was observed between biofilm mass with or without AzLys treatment, confirming that the levels of AzLys used are not inhibiting growth. The lowest AzLys concentration used in combination with E79 decreased biofilm mass 1.7-fold as compared to E79 alone (*p* < 0.01), while the highest AzLys PAS concentration showed a 3.1-fold decrease as compared to phage treatment alone (*p* < 0.0001). No statistically significant difference between the biofilm mass for the four PAS treatment groups was observed. These results show that AzLys increases the biofilm destruction activity of E79 phage, with combination of the two treatments resulting in a significant reduction in biofilm mass compared to phage treatment alone.

### 3.5. Effects of Sub-Inhibitory AzLys on PA01 Surface Structures

**Figure 6:**
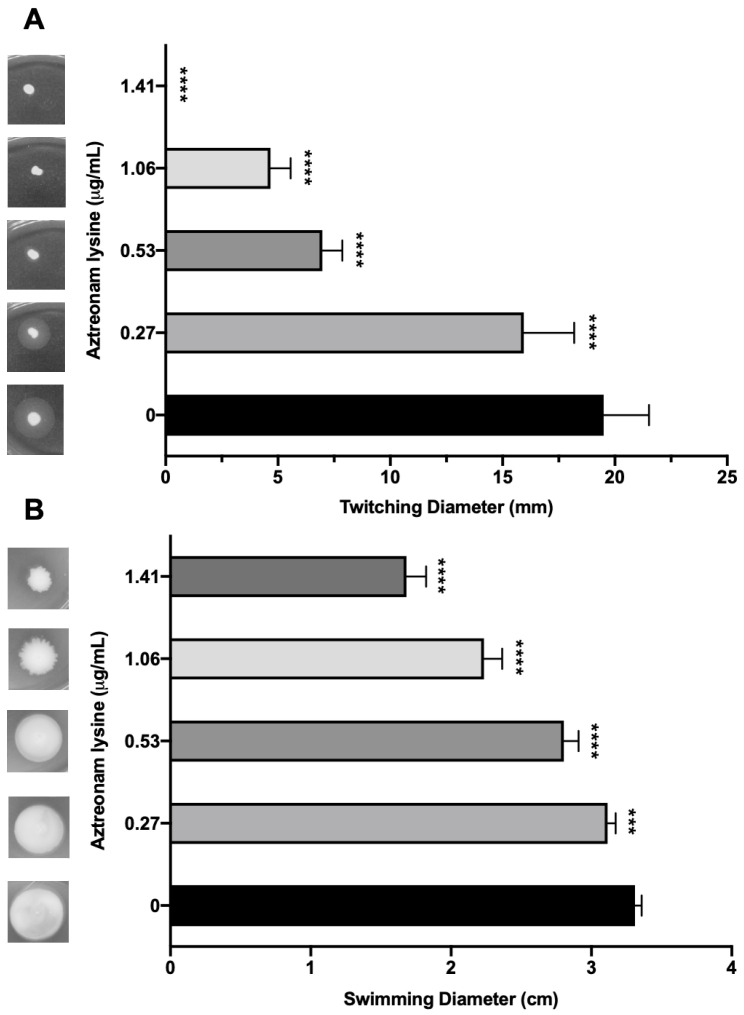
Effect of sub-inhibitory concentrations of aztreonam lysine (AzLys) on the motility of *P. aeruginosa* PA01. (**A**) PA01 grown in the presence or absence of sub-inhibitory AzLys concentrations were stab inoculated through 1% agar and incubated for 24 h at 37 °C. Twitching zones below the agar were visualized with TM developer solution and the diameter was measured using ImageJ [29]. (**B**) PA01 grown in the presence or absence of sub-inhibitory AzLys concentrations were stab inoculated midway through 0.3% ½ LB agar and incubated for 24 h at 37 °C. Diameter of swimming zone was measured using ImageJ. Representative twitching or swimming zones are shown on the left and the average diameter of each from nine replicates is shown on the right, including error bars showing standard deviation. Statistical analyses were performed using ANOVA and Dunnett’s post-test compared to the controls (***, *p* < 0.001; ****, *p* < 0.0001).

Though AzLys can increase the activity of a phage that uses LPS as its receptor, it seems unlikely that it would be able to increase the activity of a phage that uses a receptor whose expression might decrease due to filamentation, rather than increase like LPS. Numerous studies suggest that sub-inhibitory concentrations of cell wall disrupting antibiotics may negatively affect bacterial virulence factors [55,56,57,58,59,60]. Due to the changed cellular morphology of PA01 caused by the presence of sub-inhibitory AzLys, we were interested to determine whether polar motility structures on the cell surface such as pili and flagella were also affected [61]. Type IV pili (T4P) are cell surface structures involved in cell motility, virulence, and adhesion [62], and are a common receptor for many *P. aeruginosa* specific phages [27]. Based on the observation that PA01 undergoes aberrant cell division in the presence of AzLys, resulting in linked, filamentous cells and a reduced number of poles, it is likely that T4P expression or function may be decreased under these conditions. Assessment of T4P function via twitching motility for PA01 bacteria grown in the presence of four different sub-inhibitory AzLys concentrations revealed that sub-inhibitory levels of AzLys significantly decreases twitching motility in a concentration-dependent manner (Figure 6A). All four AzLys concentrations tested show a significant decrease in twitching motility compared to the twitching diameter of 19.5 ± 2.0 mm when PA01 is grown in the absence of AzLys. Twitching motility was eliminated at the highest sub-inhibitory AzLys concentration of 1.41 μg/mL. These results show that sub-inhibitory levels of AzLys have a concentration-dependent negative effect on T4P function in PA01.

In addition to T4P, we investigated whether AzLys also negatively affects the function of other polar motility structures. *P. aeruginosa* has a single polar flagellum [63] that is responsible for mediating bacterial movement through liquid or low-viscosity conditions, known as swimming motility [32]. If aberrantly divided filamentous cells express fewer flagella based on cell mass than normal, the addition of AzLys may result in decreased swimming ability. Assessment of swimming motility in the presence of sub-inhibitory concentrations of AzLys shows a decrease in swimming zone diameter (Figure 6B). This negative effect is concentration dependent, with the highest concentration of 1.41 μg/mL reducing flagellar function by approximately 49.3% compared to cells grown without AzLys. While this concentration-dependent effect is similar to the observed reduction in twitching motility, 1.41 μg/mL AzLys did not eliminate swimming motility as it did for twitching motility (Figure 6).

Fonseca et al. [58] similarly observed a decrease in both twitching and swimming motility following the exposure of eight different strains of *P. aeruginosa* to 0.5 MIC piperacillin/tazobactam. However, antibiotics can also have the opposite effect on motility; an increase in swimming motility was observed for *Chromobacterium violaceum* when exposed to sub-inhibitory concentrations of kanamycin [64]. How an antibiotic affects motility and other virulence factors is likely determined by the way the cell responds to the environmental stressor. Therefore, it may be important to pair a cell’s response to an antibiotic with the requirements of the phage infection cycle when searching for synergistic activity between an antibiotic and a phage. It is plausible that *P. aeruginosa* phages using the T4P or flagella as a receptor may not exhibit a significant increase in phage adsorption or infection efficiency in the presence of antibiotics that cause filamentation, and PAS effects may not be observed.

### 3.6. Effects of AzLys on T4P-Specific Phage phiKZ

**Figure 7:**
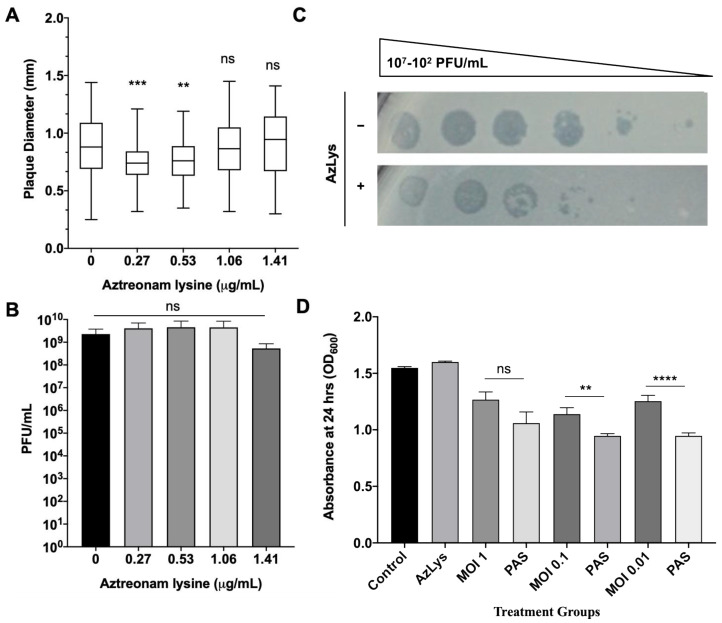
Effects of aztreonam lysine (AzLys) on *P. aeruginosa* phage phiKZ that uses the T4P as its receptor. (**A**) Box plot of plaque diameter, and (**B**) bar chart of average phage titer, of phage phiKZ in the presence of different concentrations of sub-inhibitory AzLys determined by a modified double agar overlay assay. Results are from three separate trials, with error bars representing the (**A**) largest and smallest plaque diameter and (**B**) standard deviation. Statistical analysis was performed using a one-way ANOVA with Dunnett’s post-test compared to the control (** *p* < 0.01; *** *p* < 0.001; ns, not significant). (**C**) Infection efficiency of phiKZ in the absence (-) or (+) presence of 1.06 μg/mL AzLys. Images are representative of three biological replicates, each with three technical replicates. (**D**) Killing effect of phage phiKZ was examined with or without the addition of 1.06 μg/mL AzLys. Values are averages from three trials, with error bars representing the standard error of the mean (SEM). Statistical analysis was performed using unpaired *t*-tests (** *p* < 0.01, **** *p* < 0.0001; ns, not significant).

To test the hypothesis that filamentation may not increase the activity of phages that use receptors found at the poles of *P. aeruginosa*, we examined the plaque diameter and phage titer of phiKZ in the presence of varying sub-inhibitory concentrations of AzLys. The virulent phage phiKZ requires a functional T4P to infect *P. aeruginosa* [65]. Our results show that AzLys did not cause a significant increase in phiKZ plaque diameter or phage titer compared to the control (Figure 7A,B), and a decrease in plaque size at the two lowest sub-inhibitory concentrations is observed (*p* < 0.001, *p* < 0.01). A possible explanation for this decrease in plaque size could be reduced phage adsorption rates due to reduced access to cellular receptors caused by filamentation. It is unknown why the plaques returned to average size as AzLys is increased, but it is possible that phiKZ exhibits a decreased latency period and/or accelerated time to lysis, as seen with E79, allowing the plaques to return average size as the effects of the antibiotics are increased. 

To examine whether phiKZ efficiency of infection is affected by AzLys, phiKZ was spotted in 10-fold dilutions on double agar overlays with and without 1.06 μg/mL AzLys (Figure 7C). PhiKZ efficiency of infection is decreased by approximately 10-fold, and zones of clearing look more diffuse in the presence of AzLys. To further investigate how cell exposure to AzLys affects the activity of phages that use T4P as their receptor, we assessed whether phiKZ exhibits a change in killing activity in the presence of 1.06 μg/mL AzLys (Figure 7D). PhiKZ was added at MOIs of 1, 0.1, and 0.01 to exponential-phase cells and optical density (OD_600_) was measured at 24 h. Unexpectedly, PA01 exhibited a greater reduction in growth with combination treatment as compared to phiKZ treatment alone, at all MOIs, two of which were statistically significant (MOI 0.1, *p* < 0.01; MOI 0.01, *p* < 0.0001). These results indicate that although there was no observed increase in phiKZ plaque size, phage production, or infection efficiency, we were still able to observe PAS with AzLys on PA01.

### 3.7. Effects of AzLys on PhiKZ Phage Growth Curve

**Figure 8:**
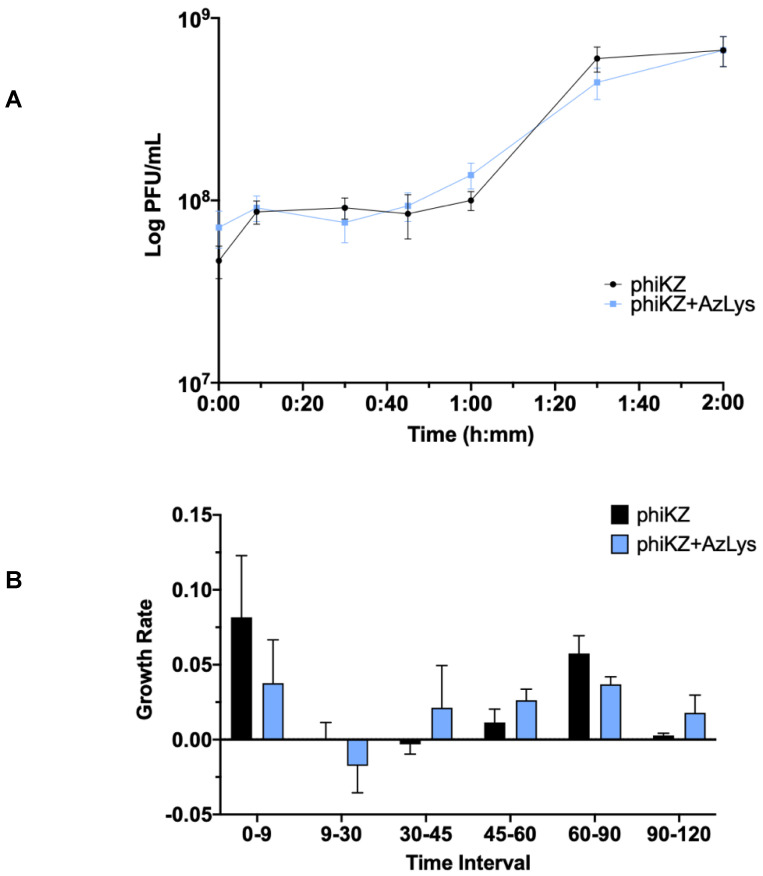
Aztreonam lysine (AzLys) shortens the eclipse period of the phiKZ one-step growth curve. Phage phiKZ was mixed with *P. aeruginosa* PA01 grown with or without 1.06 μg/mL AzLys at a MOI of approximately three and phage titer was determined periodically over a 2 h incubation. (**A**) The one-step growth curve of phiKZ, with each point representing the average of nine samples from three separate trials. Statistical analysis was performed using a paired *t*-test. (**B**) Growth rate of graph in (**A**) determined using the averages from each trial and calculated using growth rate equation log10N-log10N0 = (μ/2.303) (t-t0). All error bars represent the standard error of the mean (SEM).

Due to the increase in killing action observed by combination treatment with phiKZ and AzLys, we sought to determine how AzLys might affect the growth curve of phiKZ. We performed a one-step phage growth curve for phiKZ on PA01 in the presence or absence of 1.06 μg/mL AzLys. Similar to the trend seen in the E79 growth curves, the phiKZ curves differ from each other in a number of ways, but some time points remain remarkably similar between the two groups (Figure 8A). In both curves, the phiKZ titer rises slightly at 9 min, and no obvious decrease in titer is observed (Figure 8A,B). Thus, we were unable to calculate phage adsorption rates. Though this is unfortunate, it is not uncommon for some one-step growth curves to lack an obvious adsorption phase [13,17,40]. Moderate accelerated time to lysis in the presence of AzLys occurs from 30 to 60 min, where phiKZ is being released both earlier and at a faster rate, as compared to the control (Figure 8A,B). Similar to the E79 growth curves, after the accelerated lysis, phage production begins to slow, and phiKZ in the presence of AzLys completes its cycle at the same time as the control, with no obvious increase in final phage production (Figure 8A,B). We also see a small decrease in burst size from 14 to 9 in the presence of AzLys. However, no significant decrease in phage titer after overnight infection was observed for phiKZ (Figure 7B). The phenomenon of accelerated time to lysis has been observed before with penicillin, another cell wall inhibiting antibiotic, and was shown to accelerate time to lysis for a staphylococcus phage [41]. As discussed previously, Kim et al. [17] showed contrasting results with other antibiotics that cause filamentation of *E. coli*, resulting in a delayed time to lysis and increased phage production. A plausible explanation for how accelerated time to lysis paired with a slight reduction in burst size causes increased activity is that accelerated phage maturation allows for phage to be released and infect new cells faster, accelerating the overall rate of killing. These results together suggest that the phenomenon of PAS is more complex than just a change in cell morphology that causes a consistent change in phage activity, and likely depends on a variety of factors including the type of bacteria, phage, and antibiotic being used.

## 4. Conclusions

It is estimated that 60–80% of CF adults will develop a chronic *P. aeruginosa* lung infection [2]. Once *P. aeruginosa* progresses to a chronic infection in individuals with CF, eradication of these infections is nearly impossible [66]. In addition, re-colonization is common due to *P. aeruginosa* persistence in the upper airways [66]. However, PAS has the potential to improve treatment for chronic infections with the combined action of antibiotics and select phages increasing killing activity against pathogens [13,14,16,36,37,41,45,46,47,48,49,50,51,52,53,67,68,69,70,71,72,73,74,75], and a variety of studies have shown that the combined use of antibiotics and phages reduces the emergence of resistance to one or both killing agents [16,68,69,70,74,75,76,77]. In this study, we show that AzLys is able to increase the activity of phages E79 and phiKZ. Phage E79 has previously been shown to be a promising candidate for use in phage therapy [21] and its activity appears to be increased by a combination of increased adsorption to the host, increased infection efficiency, and accelerated time to lysis. This study also shows that although AzLys negatively affects the expression of the T4P virulence factor, an increased killing activity of T4P-dependent phiKZ was observed that could possibly be caused by accelerated time to lysis. This finding highlights the hypothesis that PAS is complex and occurs through a variety of different mechanisms. It further shows that standard methods for identifying PAS, such as plaque size and phage titer, are not always sufficient to determine whether synergy exists. Overall, we demonstrate the efficacy of PAS against *P. aeruginosa* PA01 in vitro and help to establish PAS with AzLys as a promising treatment expansion for the CF community.

## Data Availability

Data sharing not applicable.

## Supplementary Materials

The following are available online at https://www.mdpi.com/2076-2607/9/1/152/s1, Figure S1: MIC of aztreonam lysine (AzLys) and aztreonam on *P. aeruginosa* PA01. Figure S2: MIC of aztreonam lysine (AzLys) on different *P. aeruginosa* strains. Table S1: AzLys MICs across *P. aeruginosa* strains.

## References

[1] Pachori P., Gothalwal R., Gandhi P. (2019). Emergence of antibiotic resistance *Pseudomonas aeruginosa* in intensive care unit; a critical review. Genes Dis..

[2] Parkins M.D., Somayaji R., Waters V.J. (2018). Epidemeology, biology, and impact of clonal *Pseudomonas aeruginosa* infections in cystic fibrosis. Clin. Microbiol. Rev..

[3] Cystic Fibrosis Foundation (2019). Cystic Fibrosis Foundation Patient Registry 2018. Annual Data Report.

[4] Moradali M.F., Ghods S., Rehm B.H.A. (2017). *Pseudomonas aeruginosa* lifestyle: A paradigm for adaptation, survival, and persistence. Front. Cell. Infect. Microbiol..

[5] Cutting G.R. (2014). Cystic fibrosis genetics: From molecular understanding to clinical application. Nat. Rev. Genet..

[6] Gellatly S.L., Hancock R.E.W. (2013). *Pseudomonas aeruginosa*: New insights into pathogenesis and host defenses. Pathog. Dis..

[7] Matsui H., Grubb B.R., Tarran R., Randell S.H., Gatzy J.T., Davis C.W., Boucher R.C. (1998). Evidence for periciliary liquid layer depletion, not abnormal ion composition, in the pathogenesis of cystic fibrosis airways disease. Cell.

[8] Scoffone V.C., Chiarelli L.R., Trespidi G., Mentasti M., Riccardi G., Buroni S. (2017). *Burkholderia cenocepacia* infections in cystic fibrosis patients: Drug resistance and therapeutic approaches. Front. Microbiol..

[9] Semler D.D., Lynch K.H., Dennis J.J. (2012). The promise of bacteriophage therapy for *Burkholderia cepacia* complex respiratory infections. Front. Cell. Infect. Microbiol..

[10] O’Neill J. (2016). Tackling Drug-Resistant Infections Globally: Final Report and Recommendations. The Review on Antimicrobial Resistance. http://amr-review.org/Publications.

[11] Loc-Carrillo C., Abedon S.T. (2011). Pros and cons of phage therapy. Bacteriophage.

[12] Abedon S.T., Kuhl S.J., Blasdel B.G., Kutter E.M. (2011). Phage treatment of human infections. Bacteriophage.

[13] Comeau A.M., Tétart F., Trojet S.N., Prère M.-F., Krisch H.M. (2007). Phage-Antibiotic Synergy (PAS): β-lactam and quinolone antibiotics stimulate virulent phage growth. PLoS ONE.

[14] Kamal F., Dennis J.J. (2015). *Burkholderia cepacia* complex Phage-Antibiotic Synergy (PAS): Antibiotics stimulate lytic phage activity. Appl. Environ. Microbiol..

[15] Ryan E.M., Alkawareek M.Y., Donnelly R.F., Gilmore B.F. (2012). Synergistic phage-antibiotic combinations for the control of *Escherichia coli* biofilms in vitro. FEMS Immunol. Med. Microbiol..

[16] Chaudhry W.N., Concepción-Acevedo J., Park T., Andleeb S., Bull J.J., Levin B.R. (2017). Synergy and order effects of antibiotics and phages in killing *Pseudomonas aeruginosa* biofilms. PLoS ONE.

[17] Kim M., Jo Y., Hwang Y.J., Hong H.W., Hong S.S., Park K., Myung H. (2018). Phage-antibiotic synergy via delayed lysis. Appl. Environ. Microbiol..

[18] Torres-Barceló C., Hochberg M.E. (2016). Evolutionary rationale for phages as complements of antibiotics. Trends Microbiol..

[19] (2018). Wanted: A reward for antibiotic development. Nat. Biotechnol..

[20] Hutchinson D., Barclay M., Prescott W.A., Brown J. (2013). Inhaled aztreonam lysine: An evidence-based review. Expert Opin. Pharmacother..

[21] Trend S., Chang B.J., O’Dea M., Stick S.M., Kicic A. (2018). Use of a primary epithelial cell screening tool to investigate phage therapy in cystic fibrosis. Front. Pharmacol..

[22] Holloway B.W. (1955). Genetic recombination in *Pseudomonas aeruginosa*. Microbiology.

[23] Holloway B.W., Egan J.B., Monk M. (1960). Lysogeny in *Pseudomonas aeruginosa*. Aust. J. Exp. Biol. Med. Sci..

[24] Slayter H.S., Holloway B.W., Hall C.E. (1964). The structure of *Pseudomonas aeruginosa* phages B3, E79, and F116. J. Ultrastruct. Res..

[25] Krylov V.N., Zhazykov I.Z. (1978). *Pseudomonas* bacteriophage phiKZ—Possible model for studying the genetic control of morphogenesis. Genetika.

[26] Kropinski A.M., Mazzocco A., Waddell T.E., Lingohr E., Johnson R.P. (2009). Enumeration of bacteriophages by double agar overlay plaque assay. Methods Mol. Biol..

[27] McCutcheon J.G., Peters D.L., Dennis J.J. (2018). Identification and characterization of type IV pili as the cellular receptor of broad host range *Stenotrophomonas maltophilia* bacteriophages DLP1 and DLP2. Viruses.

[28] Wiegand I., Hilpert K., Hancock R.E.W. (2008). Agar and broth dilution methods to determine the minimal inhibitory concentration (MIC) of antimicrobial substances. Nat. Protoc..

[29] Schneider C., Rasband W., Eliceiri K. (2012). NIH Image to ImageJ: 25 years of image analysis. Nat. Methods.

[30] Carlson K., Kutter E., Sulakvelidze A. (2004). Appendix: Working with bacteriophages: Common techniques and methodological approaches. Bacteriophages: Biology and Applications.

[31] Turnbull L., Whitchurch C.B. (2014). Motility assay: Twitching motility. Methods Mol. Biol..

[32] Ha D.-G., Kuchma S.L., O’Toole G.A. (2014). Plate-based assay for swimming motility in *Pseudomonas aeruginosa*. Methods Mol. Biol..

[33] Bray D.F., Bagu J., Koegler P. (1993). Comparison of hexamethyldisilazane (HMDS), Peldri II, and critical-point drying methods for scanning electron microscopy of biological specimens. Microsc. Res. Tech..

[34] Yamagami H., Endo H. (1969). Loss of lysis inhibition in filamentous *Escherichia coli* infected with wild-type bacteriophage T4. J. Virol..

[35] Santos S.B., Carvalho C.M., Sillankorva S., Nicolau A., Ferreira E.C., Azeredo J. (2009). The use of antibiotics to improve phage detection and enumeration by the double-layer agar technique. BMC Microbiol..

[36] Knezevic P., Curcin S., Aleksic V., Petrusic M., Vlaski L. (2013). Phage-antibiotic synergism: A possible approach to combatting *Pseudomonas aeruginosa*. Res. Microbiol..

[37] Hadas H., Einav M., Fishov I., Zaritsky A. (1997). Bacteriophage T4 development depends on the physiology of its host *Escherichia coli*. Microbiology.

[38] Jarrell K., Kropinski A.M. (1977). Identification of the cell wall receptor for bacteriophage E79 in *Pseudomonas aeruginosa* strain PAO. J. Virol..

[39] Kaur S., Harjai K., Chhibber S. (2012). Methicillin-resistant *Staphylococcus aureus* phage plaque size enhancement using sublethal concentrations of antibiotics. Appl. Environ. Microbiol..

[40] Abedon S.T., Herschler T.D., Stopar D. (2001). Bacteriophage latent-period evolution as a response to resource availability. Appl. Environ. Microbiol..

[41] Krueger A.P., Cohn T., Smith P.N., Mcguire C.D. (1948). Observations on the effect of penicillin on the reaction between phage and staphylococci. J. Gen. Physiol..

[42] Price W.H. (1947). Bacteriophage formation without bacterial growth; the effect of niacin and yeast extract on phage formation and bacterial growth in the presence of penicillin. J. Gen. Physiol..

[43] Price W.H. (1947). Bacteriophage formation without bacterial growth; formation of staphylococcus phage in the presence of bacteria inhibited by penicillin. J. Gen. Physiol..

[44] Price W.H. (1947). Bacteriophage formation without bacterial growth; the effect of iodoacetate, fluoride, gramicidin, and azide on the formation of bacteriophage. J. Gen. Physiol..

[45] Akturk E., Oliveira H., Santos S.B., Costa S., Kuyumcu S., Melo L.D.R., Azeredo J. (2019). Synergistic action of phage and antibiotics: Parameters to enhance the killing efficacy against mono and dual-species biofilms. Antibiotics.

[46] Chang R.Y.K., Das T., Manos J., Kutter E., Morales S., Chan H.K. (2019). Bacteriophage PEV20 and ciprofloxacin combination treatment enhances removal of *Pseudomonas aeruginosa* biofilm isolated from cystic fibrosis and wound patients. AAPS J..

[47] Coulter L.B., McLean R.J.C., Rohde R.E., Aron G.M. (2014). Effect of bacteriophage infection in combination with tobramycin on the emergence of resistance in *Escherichia coli* and *Pseudomonas aeruginosa* biofilms. Viruses.

[48] Henriksen K., Rørbo N., Rybtke M.L., Martinet M.G., Tolker-Nielsen T., Høiby N., Middelboe M., Ciofu O. (2019). *P. aeruginosa* flow-cell biofilms are enhanced by repeated phage treatments but can be eradicated by phage-ciprofloxacin combination. Pathog. Dis..

[49] Moulton-Brown C.E., Friman V.P. (2018). Rapid evolution of generalized resistance mechanisms can constrain the efficacy of phage–antibiotic treatments. Evol. Appl..

[50] Nouraldin A.A.M., Baddour M.M., Harfoush R.A.H., Essa S.A.M. (2016). Bacteriophage-antibiotic synergism to control planktonic and biofilm producing clinical isolates of *Pseudomonas aeruginosa*. Alexandria J. Med..

[51] Tkhilaishvili T., Winkler T., Müller M., Perka C., Trampuz A. (2020). Bacteriophages as adjuvant to antibiotics for the treatment of periprosthetic joint infection caused by multidrug-resistant *Pseudomonas aeruginosa*. Antimicrob. Agents Chemother..

[52] Tkhilaishvili T., Wang L., Perka C., Trampuz A., Gonzalez Moreno M. (2020). Using bacteriophages as a trojan horse to the killing of dual-species biofilm formed by *Pseudomonas aeruginosa* and methicillin resistant *Staphylococcus aureus*. Front. Microbiol..

[53] Uchiyama J., Shigehisa R., Nasukawa T., Mizukami K., Takemura-Uchiyama I., Ujihara T., Murakami H., Imanishi I., Nishifuji K., Sakaguchi M. (2018). Piperacillin and ceftazidime produce the strongest synergistic phage–antibiotic effect in *Pseudomonas aeruginosa*. Arch. Virol..

[54] Donlan R.M. (2002). Biofilms: Microbial life on surfaces. Emerg. Infect. Dis..

[55] Braga P.C., Sasso M.D., Sala M.T. (2000). Sub-MIC concentrations of cefodizime interfere with various factors affecting bacterial virulence. J. Antimicrob. Chemother..

[56] De Andrade J.P.L., de Macêdo Farias L., Ferreira J.F.G., Bruna-Romero O., da Glória de Souza D., de Carvalho M.A.R., dos Santos K.V. (2016). Sub-inhibitory concentration of piperacillin–tazobactam may be related to virulence properties of filamentous *Escherichia coli*. Curr. Microbiol..

[57] Drago L., De Vecchi E., Mombelli B., Nicola L., Valli M., Gismondo M.R. (2001). Activity of levofloxacin and ciprofloxacin against urinary pathogens. J. Antimicrob. Chemother..

[58] Fonseca A.P., Extremina C., Fonseca A.F., Sousa J.C. (2004). Effect of subinhibitory concentration of piperacillin/tazobactam on *Pseudomonas aeruginosa*. J. Med. Microbiol..

[59] Fonseca A.P., Sousa J.C. (2007). Effect of antibiotic-induced morphological changes on surface properties, motility and adhesion of nosocomial *Pseudomonas aeruginosa* strains under different physiological states. J. Appl. Microbiol..

[60] Wolter J.M., McCormack J.G. (1998). The effect of subinhibitory concentrations of antibiotics on adherence of *Pseudomonas aeruginosa* to cystic fibrosis (CF) and non-CF-affected tracheal epithelial cells. J. Infect..

[61] Bradley D.E. (1980). A function of *Pseudomonas aeruginosa* PAO polar pili: Twitching motility. Can. J. Microbiol..

[62] Burrows L.L. (2012). *Pseudomonas aeruginosa* twitching motility: Type IV pili in action. Annu. Rev. Microbiol..

[63] Campodónico V.L., Llosa N.J., Grout M., Döring G., Maira-Litrán T., Pier G.B. (2010). Evaluation of flagella and flagellin of *Pseudomonas aeruginosa* as vaccines. Infect. Immun..

[64] Liu Z., Wang W., Zhu Y., Gong Q., Yu W., Lu X. (2013). Antibiotics at subinhibitory concentrations improve the quorum sensing behavior of *Chromobacterium violaceum*. FEMS Microbiol. Lett..

[65] Danis-Wlodarczyk K., Vandenheuvel D., Jang H.B., Briers Y., Olszak T., Arabski M., Wasik S., Drabik M., Higgins G., Tyrrell J. (2016). A proposed integrated approach for the preclinical evaluation of phage therapy in *Pseudomonas* infections. Sci. Rep..

[66] Sousa A.M., Pereira M.O. (2014). *Pseudomonas aeruginosa* diversification during infection development in cystic fibrosis lungs—A review. Pathogens.

[67] Chhibber S., Kaur T., Kaur S. (2013). Co-therapy using lytic bacteriophage and linezolid: Effective treatment in eliminating methicillin resistant *Staphylococcus aureus* (MRSA) from diabetic foot infections. PLoS ONE.

[68] Jo A., Ding T., Ahn J. (2016). Synergistic antimicrobial activity of bacteriophages and antibiotics against *Staphylococcus aureus*. Food Sci. Biotechnol..

[69] Jo A., Kim J., Ding T., Ahn J. (2016). Role of phage-antibiotic combination in reducing antibiotic resistance in *Staphylococcus aureus*. Food Sci. Biotechnol..

[70] Kirby A.E. (2012). Synergistic action of gentamicin and bacteriophage in a continuous culture population of *Staphylococcus aureus*. PLoS ONE.

[71] Lin Y., Chang R.Y.K., Britton W.J., Morales S., Kutter E., Li J., Chan H.-K. (2019). Inhalable combination powder formulations of phage and ciprofloxacin for *Pseudomonas aeruginosa* respiratory infections. Eur. J. Pharm. Biopharm..

[72] Lin Y., Chang R.Y.K., Britton W.J., Morales S., Kutter E., Chan H.K. (2018). Synergy of nebulized phage PEV20 and ciprofloxacin combination against *Pseudomonas aeruginosa*. Int. J. Pharm..

[73] Shlezinger M., Coppenhagen-Glazer S., Gelman D., Beyth N., Hazan R. (2019). Eradication of vancomycin-resistant *Enterococci* by combining phage and vancomycin. Viruses.

[74] Torres-Barceló C., Arias-Sánchez F.I., Vasse M., Ramsayer J., Kaltz O., Hochberg M.E. (2014). A window of opportunity to control the bacterial pathogen *Pseudomonas aeruginosa* combining antibiotics and phages. PLoS ONE.

[75] Verma V., Harjai K., Chhibber S. (2009). Restricting ciprofloxacin-induced resistant variant formation in biofilm of *Klebsiella pneumoniae* B5055 by complementary bacteriophage treatment. J. Antimicrob. Chemother..

[76] Chan B.K., Sistrom M., Wertz J.E., Kortright K.E., Narayan D., Turner P.E. (2016). Phage selection restores antibiotic sensitivity in MDR *Pseudomonas aeruginosa*. Sci. Rep..

[77] Zhang Q.-G., Buckling A. (2012). Phages limit the evolution of bacterial antibiotic resistance in experimental microcosms. Evol. Appl..

