# Supplementary material for: Aztreonam Lysine Increases the Activity of Phages E79 and phiKZ against Pseudomonas aeruginosa PA01
Source: Microorganisms. 2021 Jan 12;9(1):152. doi: 10.3390/microorganisms9010152 (PMC7827458; doi:10.3390/microorganisms9010152)
Supplement: Supplementary file 1 [file microorganisms-09-00152-s001.pdf]

*microorganisms*

MDPI

# Supplementary Materials: Aztreonam Lysine Increases the Activity of Phages E79 and phiKZ against *Pseudomonas aeruginosa* PA01

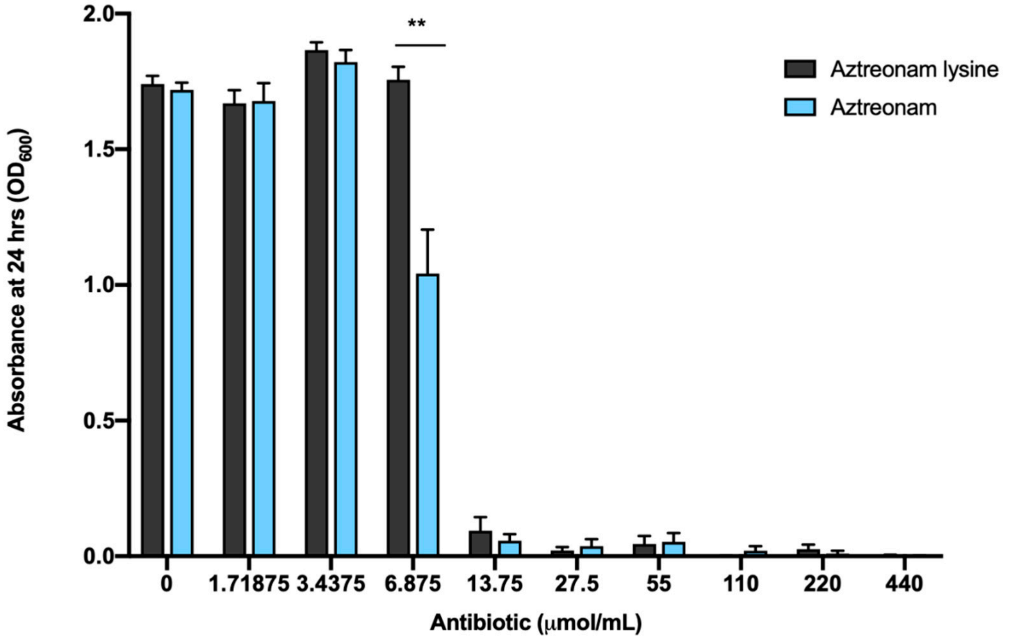


**Figure S1:** MIC of aztreonam lysine (AzLys) versus aztreonam on *P. aeruginosa* PA01. Exponential phase PA01 was diluted 1:100 to approximately $10^5$ CFU/mL and grown for 24 h at 37 °C in the presence of either AzLys or Aztreonam antibiotic at varying concentrations. Optical density at 600 nm ($OD_{600}$) was measured to obtain culture growth. Statistical analysis was performed using an unpaired t-test (**;P < 0.01). Error bars represent standard error of the mean (SEM).

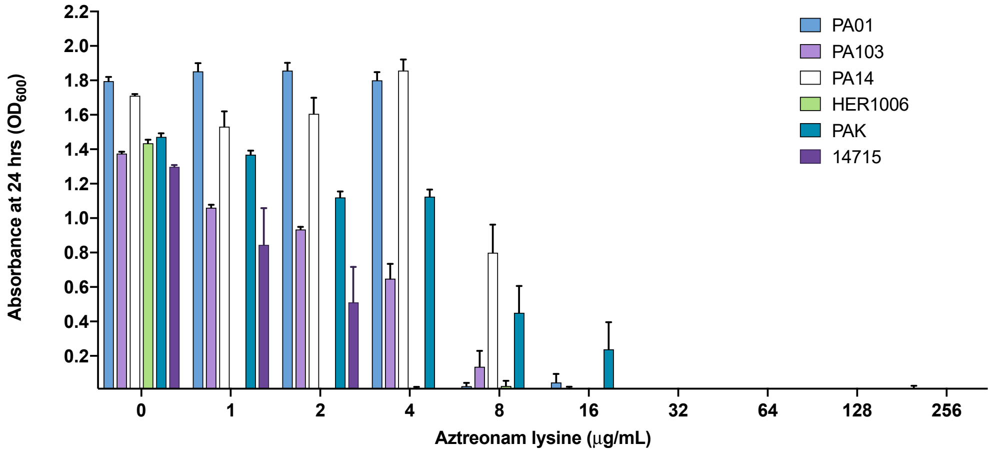


**Figure S2:** MIC of aztreonam lysine (AzLys) on different *P. aeruginosa* strains. Each strain was grown to exponential phase and diluted 1:100 to approximately $10^5$ CFU/mL and grown for 24 h at 37 °C in the presence of AzLys at varying concentrations. Optical density at 600 nm ($OD_{600}$) was measured to obtain culture growth. Error bars represent standard error of the mean (SEM).

**Citation**: Davis, C.M.; McCutcheon, J.G.; Dennis, J.J. Aztreonam Lysine Increases the Activity of Phages E79 and phiKZ against *Pseudomonas aeruginosa* PA01. *Microorganisms* **2021**, *9*, 152. https://doi.org/10.3390/microorganisms9010152

Received: 17 December 2020
Accepted: 8 January 2021
Published: 12 January 2021

**Publisher's Note:** MDPI stays neutral with regard to jurisdictional claims in published maps and institutional affiliations.

**Copyright:** © 2021 by the authors. Submitted for possible open access publication under the terms and conditions of the Creative Commons Attribution (CC BY) license (http://creativecommons.org/licenses/by/4.0/).

**Supplementary Table 1.** Aztreonam Lysine minimal inhibitory concentrations for *P. aeuruginosa* strains

| *P. aeruginosa* strain | PA01 | PA103 | PA14 | HER1006 | PAK | 14715 |
|---|---|---|---|---|---|---|
| MIC (mg/mL) | 8 | 16 | 16 | <1 | 32 | 4 |
